# Implicating effector genes at COVID-19 GWAS loci using promoter-focused Capture-C in disease-relevant immune cell types

**DOI:** 10.1186/s13059-022-02691-1

**Published:** 2022-06-03

**Authors:** Matthew C. Pahl, Carole Le Coz, Chun Su, Prabhat Sharma, Rajan M. Thomas, James A. Pippin, Emylette Cruz Cabrera, Matthew E. Johnson, Michelle E. Leonard, Sumei Lu, Alessandra Chesi, Kathleen E. Sullivan, Neil Romberg, Struan F. A. Grant, Andrew D. Wells

**Affiliations:** 1 Division of Human Genetics, The Children’s Hospital of Philadelphia, 3615 Civic Center Boulevard, Philadelphia, PA USA; 2 Department of Pathology, The Children’s Hospital of Philadelphia, 3615 Civic Center Boulevard, Philadelphia, PA USA; 3 Division of Allergy and Immunology, The Children’s Hospital of Philadelphia, 3615 Civic Center Boulevard, Philadelphia, PA USA; 4 Department of Pathology and Laboratory Medicine, Perelman School of Medicine, University of Pennsylvania, 3615 Civic Center Boulevard, Philadelphia, PA USA; 5 Department of Pediatrics, Perelman School of Medicine, University of Pennsylvania, 3615 Civic Center Boulevard, Philadelphia, PA USA; 6 Institute for Immunology, Perelman School of Medicine, University of Pennsylvania, 3615 Civic Center Boulevard, Philadelphia, PA USA; 7 Division of Diabetes and Endocrinology, The Children’s Hospital of Philadelphia, 3615 Civic Center Boulevard, Philadelphia, PA USA; 8 Department of Genetics, Perelman School of Medicine, University of Pennsylvania, 3615 Civic Center Boulevard, Philadelphia, PA USA

## Abstract

**Background:**

SARS-CoV-2 infection results in a broad spectrum of COVID-19 disease, from mild or no symptoms to hospitalization and death. COVID-19 disease severity has been associated with some pre-existing conditions and the magnitude of the adaptive immune response to SARS-CoV-2, and a recent genome-wide association study (GWAS) of the risk of critical illness revealed a significant genetic component. To gain insight into how human genetic variation attenuates or exacerbates disease following SARS-CoV-2 infection, we implicated putatively functional COVID risk variants in the cis-regulatory landscapes of human immune cell types with established roles in disease severity and used high-resolution chromatin conformation capture to map these disease-associated elements to their effector genes.

**Results:**

This functional genomic approach implicates 16 genes involved in viral replication, the interferon response, and inflammation. Several of these genes (PAXBP1, IFNAR2, OAS1, OAS3, TNFAIP8L1, GART) were differentially expressed in immune cells from patients with severe versus moderate COVID-19 disease, and we demonstrate a previously unappreciated role for GART in T cell-dependent antibody-producing B cell differentiation in a human tonsillar organoid model.

**Conclusions:**

This study offers immunogenetic insight into the basis of COVID-19 disease severity and implicates new targets for therapeutics that limit SARS-CoV-2 infection and its resultant life-threatening inflammation.

**Supplementary Information:**

The online version contains supplementary material available at 10.1186/s13059-022-02691-1.

## Background

SARS-CoV-2 induces a strong immune response dominated by CD4+ and CD8+ T cells reactive to spike antigen-derived epitopes [1, 2] and accompanied by elevated lymphokines and reduced frequencies of T and B cells in the blood. Pan-lymphopenia and higher cytokine levels are associated with severe disease [3–9], and milder disease is associated with higher frequencies of circulating SARS-CoV-2-specific CD4+ and CD8+ T cells [2, 10–13]. The lungs of COVID-19 patients are also enriched for T cells, and SARS-CoV-2-infected monocyte-derived alveolar macrophages and neutrophils producing T cell chemokines are more abundant in patients with severe disease [10, 14]. During anti-viral immune responses, CD4+ T follicular helper cells (TFH) migrate into germinal centers (GC) to help GC B cells differentiate into high affinity antibody-producing plasmablasts [15]. Circulating SARS-CoV-2-specific TFH, plasmablasts, and high-affinity Ab are detected in COVID-19 patients, and the frequency of activated TFH and plasmablasts in the blood is associated with neutralizing IgG levels [11, 16–20]. SARS-CoV-2 infection in macaques induces a similar cellular dynamic in the spleen [21, 22], and the frequency of circulating plasmablasts, naive CD4+ T cells, and TFH in humans is associated with disease severity [6, 11, 17–19, 23]. The immune dynamics of SARS-CoV-2 infection suggests that genetically encoded factors regulating the differentiation and function of CD4+ T cells, TFH, and germinal center B cells (GCB) likely influence the severity of COVID-19 disease. Recent genome-wide association studies (GWAS) for critically ill COVID-19 patients have revealed a number of loci associated with the trait [24, 25]. However, GWAS does not identify causal effector genes at non-coding signals, and these loci are often presumptively named after the nearest gene. To implicate putative causal variants and their corresponding effector genes at COVID-19 GWAS loci, we leveraged a 3D genomic variant-to-gene mapping approach using disease-relevant, human immune cell types.

## Results

**Fig. 1:**
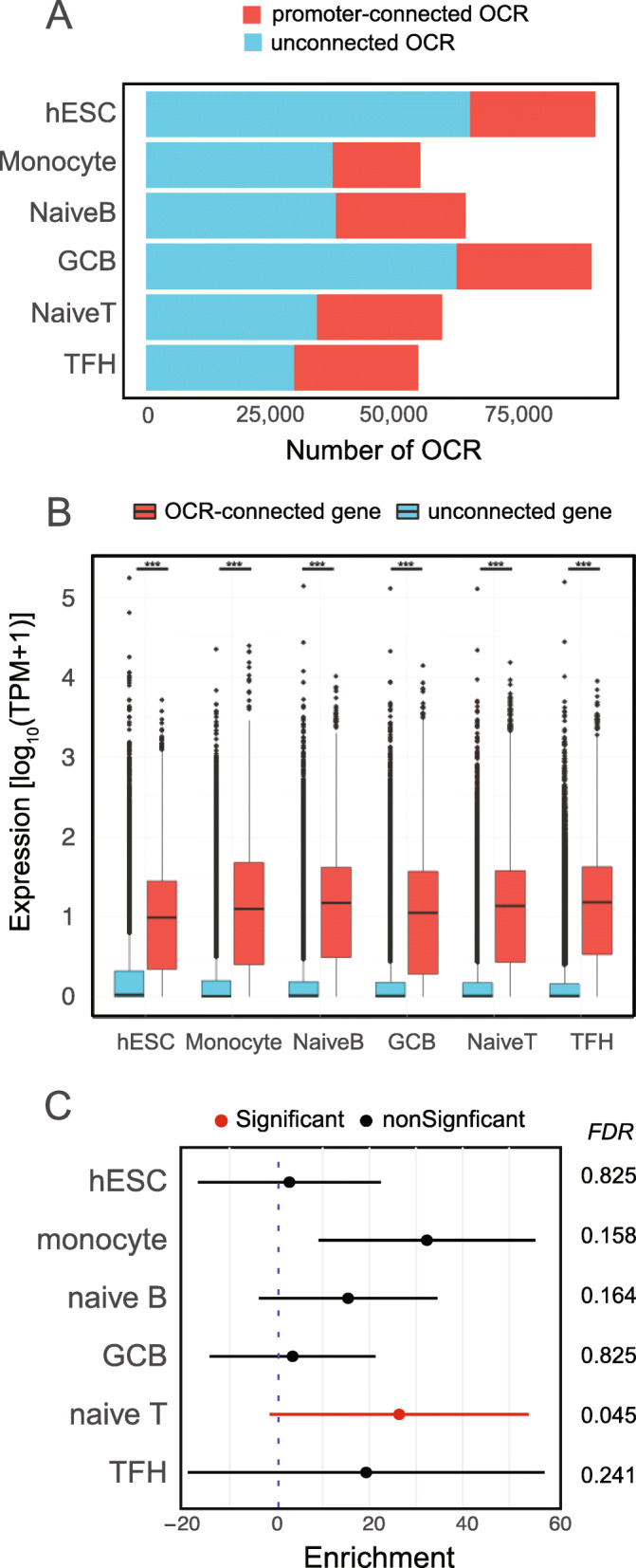
Promoter-connected open chromatin is enriched for highly expressed genes and COVID-19 disease risk heritability. **A** The number of OCRs contacting promoters determined by Capture C and those without promoter contacts. **B** Expression measured by transcripts per kilobase million (TPM) of genes with at least one OCR-promoter contact (red) vs. genes without promoter-OCR contacts (blue). Boxplots represent the median expression for each category. Statistical significance was determined using two-sided Wilcoxon rank-sum tests. TPM range contacted 0–25,499.71, non-contacted 177,277.33. Medians: hESC contacting = 8.86, non-contacting 0.0621, monocyte contacting = 11.627, non-contacting 0.0123, naïve B contacting = 14.0, non-contacting 0.0289, GCB contacting = 14.0, non-contacting 0.0289, naïve CD4 T contacting = 12.8, non-contacting 0.0244 TFH contacting = 14.3, non-contacting = 0.216. **C** Enrichment of estimated COVID-19 GWAS heritability determined by partitioned score regression for the open chromatin landscape for each cell type. Points indicate calculated enrichment and whiskers indicate 95% confidence interval. The associated FDR for each enrichment is depicted on the right

As an initial step, we used ATAC-seq to identify accessible SNPs in linkage disequilibrium (LD) with GWAS sentinel signals and high-resolution promoter-focused Capture-C (PCC) to connect them to the genes they likely regulate. Because of their connection to COVID-19 disease severity, we chose to perform these analyses in primary naive B cells, naïve CD4+ T cells, follicular helper T cells [26], and germinal center B cells from human tonsil, and circulating monocytes (Additional file 1 Fig S1A, Table S1). We included hESC as a non-immune comparator [27]. The number (range: 55k–91k open regions) and genomic distribution (mean range: 496–655 bp) of open chromatin regions (OCR) were comparable among cell types (Additional file 1 Fig S1B-E). To put these OCR in the context of the 3-dimensional structure of the genome, we performed high-resolution PCC targeting the majority of coding and non-coding genes in the human genome [26, 28–31]. The quantity and quality of promoter interactions were similar among immune cell types (Additional file 1 Fig S2, Table S2). One-third to one-half of the open chromatin landscapes were connected to gene promoters (Fig. 1A), and genes whose promoters interact with distal open chromatin regions were expressed ~ 10-fold higher than genes not physically associated with open chromatin (Fig. 1B). These results indicate that promoter-connected OCR represent *cis*-regulatory elements engaged in active control of gene expression. The promoter-connected open chromatin landscape of naive CD4+ T cells was significantly enriched for COVID-19 disease risk heritability (~ 28-fold, FDR = 0.045, Fig. 1C). TFH and monocyte gene regulatory architectures showed a similar magnitude but more variable enrichment for COVID-19 disease risk variants, and B cells and ESC landscapes did not show enrichment (Fig. 1C). These results are consistent with the growing evidence that dynamics relevant to multiple immune cell types may contribute to disease severity and suggest that COVID-19-associated variants in CD4+ T cell and monocyte open chromatin may have the strongest influence on disease severity.

**Fig. 2:**
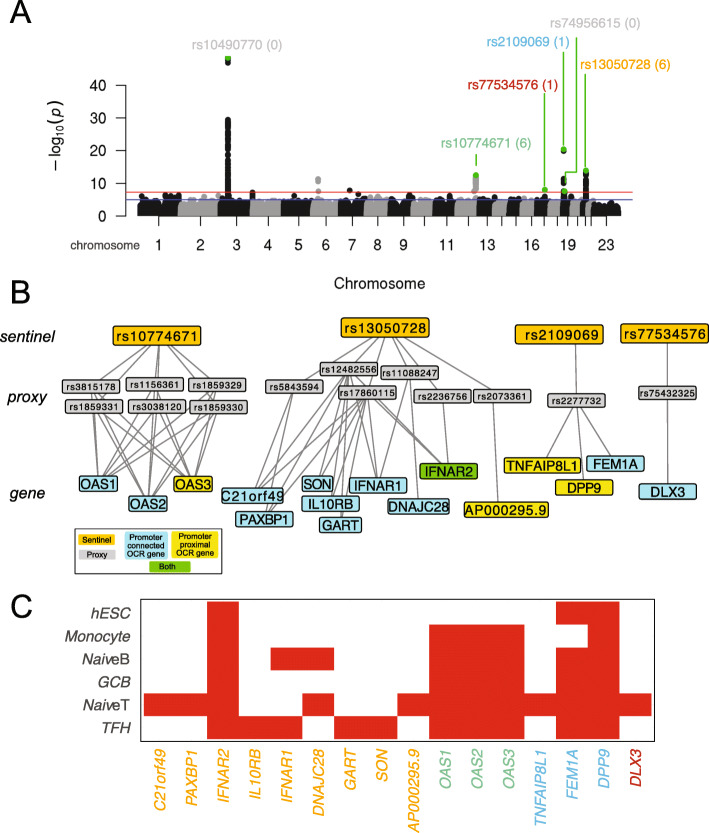
Chromosome capture-based variant-to-gene mapping identifies candidate effector genes at COVID-19 GWAS loci. **A** Manhattan plot generated using the summary statistics from the COVID-19 severity GWAS. Genome-wide significant signals are shown together with the number of accessible gene-annotated proxies associated. **B** Depiction of the statistical sentinel-proxy SNP linkages and the PCC-derived physical gene-proxy connections identified in this study. Genes in yellow were implicated by an accessible proxy in the promoter regions, genes in blue were implicated through chromatin-based contact between the promoter region and a distal accessible proxy, and green indicates implication by both promoter and distal proxies. **C** Heatmap depicting genes implicated by variant-to-gene mapping in each cell type in red. Color of each gene corresponds to the signal shown in **A**

To map potentially functional COVID-19 variants to their target genes, we intersected our ATAC-seq and PCC data with the most recent COVID-19 disease risk GWAS [25] (Fig. 2A and B, Table S3). We examined proxy SNPs in high LD (R2 > 0.8) with the six independent genome-wide significant signals associated with COVID19 severity and identified 16 genes whose promoters physically interact with accessible COVID-19-associated variants in immune cells (Fig. 2B, C, Additional file 1 Fig S3). We identified accessible proxy SNPs in LD with the COVID-19 sentinel rs10774671 in the promoter of *OAS3*, and accessible proxies interacting with *OAS1* and *OAS2* in all immune cell types analyzed (Fig. 2C). The *DPP9* sentinel rs2109069 is in LD with accessible proxies in the *DPP9* promoter in each immune cell type, but also to distal accessible proxies interacting with *FEM1A* in the T and B cells, and to distal proxies interacting with *TNFAIP8L1* specifically in naive CD4+ T cells (Fig. 2C). The sentinel rs13050728 is in LD with accessible proxies interacting with *PAXBP1*, *C21orf49*, and *AP000295.9* in naive CD4+ T cells; *IFNAR1* in TFH and naïve B cells; *DNAJC28* in naive T and B cells; *GART*, *IL10RB*, and *SON* in TFH cells; and *IFNAR2* in all cell types (Fig. 2C). A proxy SNP in LD with the sentinel rs77534576 is connected to the *DLX3* gene in naive CD4+ T cells (Fig. 2C).

Gene ontology analyses of the set of COVID-19 variant-connected genes showed enrichment for pathways involved in coronavirus pathogenesis (-logP = 8.97), viral hypercytokinemia (-logP = 8.95), viral/bacterial pattern recognition (-logP = 4.0), interferon signaling (-logP = 5.93), and T cell exhaustion (-logP = 2.39). This set of genes was also enriched (*P* < 0.0003) for factors involved in viral infection, RNA virus replication, anti-viral response, multiple sclerosis, psoriasis, and necrosis (Additional file 1 Fig S4, Table S4). We note that our approach did not implicate genes for two COVID-19-associated GWAS signals (rs10490770 & rs74956615). rs10490770 is the strongest signal located on chromosome 3, and its proxy SNP rs17713054 has been reported to contact the *LZTFL1* promoter in lung epithelial cells, suggesting that these risk loci act outside the immune cells [32].

**Fig. 3:**
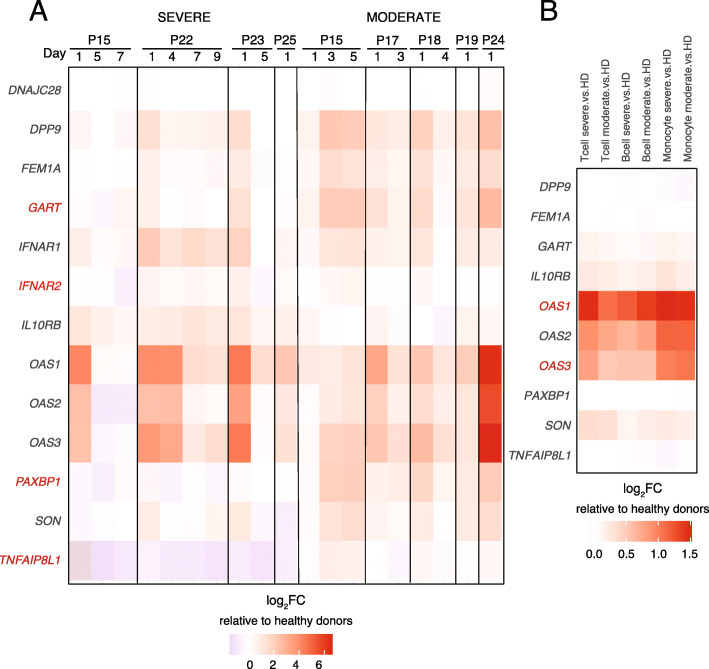
Immune genes implicated through contact with COVID-19 variants are differentially expressed in patients with SARS-CoV-2 infection and severe COVID-19 disease. Differential gene expression in mild/moderate and severe COVID-19 patients relative to healthy donors quantified by **A** bulk RNA-seq of whole blood leukocytes at various timepoints from Galani et al. [33] and **B** single-cell RNA-seq from peripheral blood from Zhang et al. [34] Values represent log_2_FC of TPM for each gene relative to the mean of healthy donors, and genes showing disease severity-associated expression are shown in red. Data in **B** represent pseudo-bulk RNA-seq of T cells, B cells, or monocytes clustered by single-cell transcript patterns. Genes indicated in red in **A** and **B** exhibited severity-associated expression patterns

To explore the functional significance of genes implicated through their connection to COVID-19-associated immune regulatory architectures, we compared their expression in bulk blood leukocytes [33] or at the single-cell level [34] from COVID-19 patients vs. healthy donors from published datasets. We found nearly two-thirds (10/16) of these implicated genes were differentially expressed in circulating leukocytes of SARS-CoV-2-infected humans (Fig. 3A, B). *OAS1, OAS2, OAS3, IL10RB, FEM1A, GART, SON,* and *IFNAR2* were significantly (FDR < 0.05) upregulated in lymphocytes and monocytes from COVID-19 patients compared to healthy controls, while *TNFAIP8L1* was significantly downregulated in monocytes from COVID-19 patients (Fig. 3A, B). Importantly, six of these genes also exhibited severity-associated patterns of expression. *IFNAR2* was significantly upregulated in severe COVID-19 patients (FDR = 0.0075, Fig. 3A), and both *OAS1* and *OAS3* were upregulated in T cells from severe COVID-19 patients compared to those with mild disease (Fig. 3B). *PAXBP1* was significantly downregulated (FDR = 0.0067) in severe vs. mild COVID patients, and *GART* and *TNFAIP8L1* were nominally downregulated in severe patients (*p* = 0.047, FDR = 0.127).

**Fig. 4:**
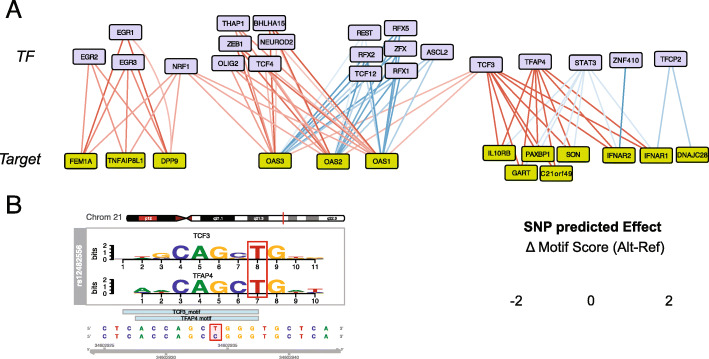
In silico prediction of transcription factor binding site disruption by accessible COVID-19 associated proxies. **A** Transcription factors (blue) with binding motifs likely to be disrupted by accessible COVID-19 SNPs and their connected target genes (green). The predicted effect of the SNP on TF binding is indicated in red for decreased affinity and in blue for increased affinity. COVID-19 risk-associated sequence variation at an element connected to *IFNAR2* is predicted to increase binding of ZNF410, a zinc finger protein involved in repression of fetal hemoglobin in erythroid cells. Risk variants also increased the predicted affinity of STAT3 for elements at six implicated genes including *PAXBP1*. Risk variants at *PAXBP1* and five other implicated genes were also predicted to reduce binding of the MYC-induced AP4 (TFAP4) oncoprotein and E2A (TCF3), a central transcription factor in lymphocyte development and malignancy. COVID-19 disease variants connected to *OAS1* and *OAS3* were predicted to affect the binding of 15 distinct transcription factors, including the E proteins TCF3, TCF4, TCF12, and NEUROD2, the RFX family of transcription factors involved in expression of a variety of immune factors including the MHC class II genes, and the plasmablast and TFH factors MIST1 (BHLHA15) and ASCL2. **B** An example of the predicted impact of the COVID-19 risk allele of rs12482556 (red) on binding of TCF3 and TFAP4

The putatively causal variants identified in this study likely influence COVID-19 risk by directly contacting and altering the expression of their target genes in immune cell types. To test whether these COVID-19 variants may influence gene transcription by affecting the binding of transcription factors, we used a modeling approach to predict their impact on the affinity of transcription factors for consensus DNA motifs present in the regulatory landscape of COVID-19-relevant immune cell types. This approach identified 9 COVID-19-associated SNPs predicted to impact binding of 22 expressed transcription factors to 14 of the 16 genes identified in this study (Fig. 4 and Table S5). COVID-19 risk-associated sequence variation at *IFNAR2* may increase binding of ZNF410, a zinc finger protein involved in repression of fetal hemoglobin in erythroid cells [35], and risk variants also increase the predicted affinity of STAT3 for elements connected to *IFNAR1*, *IFNAR2*, *PAXBP1*, *GART*, *C21ORF49*, *SON*, and *IL10RB*. Variants connected to *OAS1*, *OAS2*, and *OAS3* may affect the binding of 15 distinct transcription factors, including the RFX family of transcription factors involved in expression of MHC class II genes, and the plasmablast and TFH factors MIST1 (BHLHA15) [36] and ASCL2 [37]. This set of affected transcription factors form a network of highly co-regulated activities downstream of the TCR, particularly centered around STAT3 and EGR1 (Additional file 1 Fig S5), and enriched for roles in hematopoietic, B and T cell development, differentiation, and function (Table S6).

## Discussion

Together, our results suggest that genes central to viral genome sensing, host control of viral replication, the interferon response, and immune inflammation are likely under genetic control by common variants associated with COVID-19 disease risk. This study implicated multiple genes at all but one locus. While methods for fine-mapping GWAS signals generally assume a single causal variant acting at one effector gene, this assumption is not always valid. We observe clear evidence for pleiotropic effects of COVID-19 disease risk-associated genetic variation at the level of multiple proxies in open chromatin at each locus in the same and/or distinct cell types, and individual accessible proxies contacting multiple genes in one or across more cell types (Supplemental Figure 3). Similar pleiotropy was observed for *FTO* obesity variants on the dynamics and lineage-specific expression of distal genes such as *IRX3* and *IRX5* [38].

**Fig. 5:**
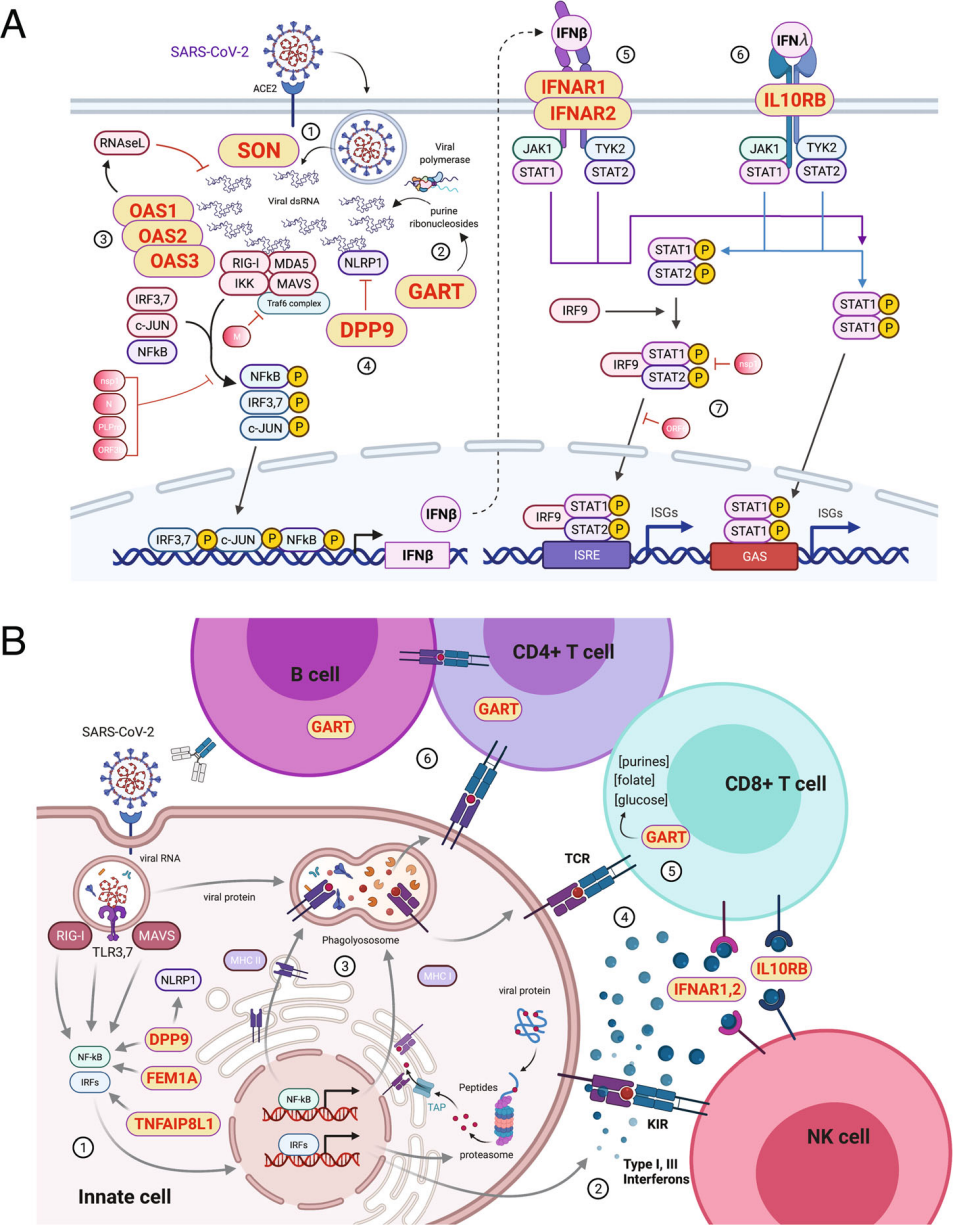
Potential mechanisms by which V2G-implicated genes impact COVID-19 disease severity. **A** SON may control release and processing of SARS-CoV-2 RNA genomes (1), and GART is involved in de novo generation of the purine precursors required for SARS-CoV-2 RNA replication (2). Sensing of dsRNA regulated by OAS1, OAS2, OAS3, and DPP9 may lead to degradation of SARS-CoV-2 genomes and activation of the inflammasome (3, 4). SARS-CoV-2-induced activation of NFkB, IRFs, and Jun, dampened by SARS-CoV-2-encoded factors (4 - M, nsp1, N, PLPro, ORF3b), induces IFNB. Anti-viral signaling is mediated by type I interferon receptors encoded by IFNAR1 and IFNAR2 (5), and the type III interferon receptor encoded by IL10RB (6). These processes are known to be inhibited by the SARS-CoV-2-encoded factors nsp1 and ORF6 (7). **B** Sensing of SARS-CoV-2 RNA genomes released upon infection of lung epithelial cells or alveolar macrophages increases expression of components of the antigen processing and presentation machinery, interferons, cytokines, and chemokines (1–3). These processes are regulated by TNFAIP8L1, FEM1A, and DPP9. Type I interferon receptors IFNAR1 and IFNAR2 control T and NK cell function, and IL10RB affects responsiveness to type III interferons and other cytokines (4). The enzymatic activity of GART regulates metabolic and epigenetic processes important for lymphocyte activation, proliferation, and differentiation (5, 6)

The genes identified through physical association with accessible COVID-19 variants have known roles in viral replication, the interferon response, and inflammation. The genes *GART* and *SON* encode factors that may directly impact SARS-CoV-2 replication (Fig. 5A). *SON* encodes a factor that regulates HBV influenza A replication [39, 40], while *GART* controls de novo purine pools required for coronavirus RNA replication that may also drive evolution of viral variants over the course of the pandemic [41, 42]. Interferons (IFN) are important for the control of early virus replication and in determining moderate vs. severe inflammatory disease [43]. SARS-CoV2 induces type I and type III interferons [44] that signal through *IFNAR1*, *IFNAR2*, and *IL10RB* (Fig. 5A), but SARS-CoV2 also encodes factors that can inhibit type I and III responses [45–47]. Thus, many SARS-CoV-2-infected individuals exhibit blunted and/or delayed interferon responses [20, 48–50] and experience more severe disease than COVID-19 patients with strong interferon responses [32, 50, 51]. SARS-CoV-2 dsRNA genomes are sensed by the RIG-I/MDA5 and RNAseL pathways [52, 53]. *OAS1*, *OAS2*, and *OAS3* encode crucial regulators of dsRNA degradation by RNAseL, and *DPP9* regulates the activity of NLRP1, a dsRNA-sensing component of the inflammasome [54] (Fig. 5A). Gain of function mutations in *OAS1* lead to autoinflammatory disease in humans [55]; polymorphisms at the *OAS1* locus are associated with type 2 diabetes [56], a pre-existing condition associated with severe COVID-19 disease [57]; and genetic variation at *DPP9* is associated with the risk of developing pulmonary fibrosis [58]. Cytokine release syndrome is a major inflammatory complication in patients with severe COVID-19 disease [59–61]. Receptors for type I (*IFNAR1* and *2*) and III (*IL10RB*) interferons drive inflammation mediated by NK and CD8+ T cells, and IL-10RB binds IL-10 whose levels are a severity predictor in COVID-19 [62] (Fig. 5B). *FEM1A* encodes a negative regulator of NFkB activation [63], and TNFAIP8L1 regulates expression of the chemokine MCP-1 [64]. *DNAJC28* is a mitochondrial Hsp40 family member and cofactor of Hsp70 heat shock proteins [65]. *PAXBP1* encodes a regulator of ROS and p53 [66], and *DLX3* encodes a homeobox protein known to function downstream of the TGFB, BMP, and WNT pathways in tooth and placental development [67], but immune roles for these factors have not been established.

**Fig. 6:**
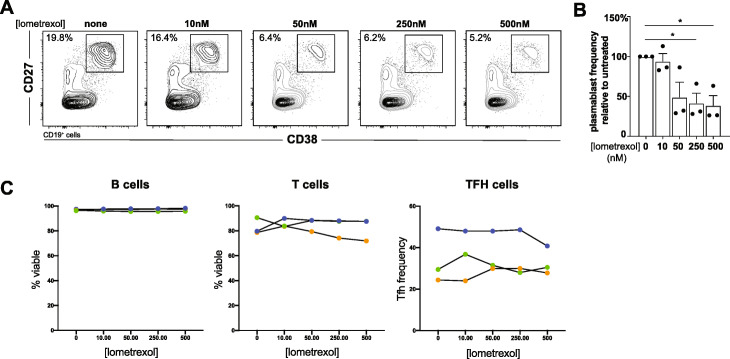
GART inhibition abrogates germinal center plasmablast output in tonsillar organoids. **A** Day 7 plasmablast frequencies from untreated or lometrexol-treated tonsillar organoids from a representative tonsil donor. **B** Plasmablast frequency diminishment in day 7 lometrexol drugged organoids relative to untreated counterparts from three tonsil donors. *P ≤ 0.05. **C** B and T cell viability and TFH frequency from these same experiments. Data from each donor are depicted separately

*GART* encodes an enzyme involved in purine biosynthesis, and its folate-derived metabolites have roles in DNA methylation and mitochondrial redox, processes that regulate immune cell function [68] (Fig. 5B). To test for a role for GART in adaptive immune responses associated with susceptibility to severe COVID-19, we used the GART inhibitory drug lometrexol in an in vitro human tonsillar organoid model of T cell-dependent germinal center B cell differentiation [32]. After 7 days in culture, T-B interactions in control organoids supported the differentiation of CD27 + CD38+ GCB cell plasmablasts (Fig. 6A and B) capable of producing high-affinity class-switched antibodies in this mod [32]. The GART inhibitor lometrexol abrogated plasmablast differentiation in a dose-dependent manner (Fig. 6A and B) without affecting B or T cell survival or TFH frequency (Fig. 6C). These results indicate that GART has a previously unappreciated role in T cell-B cell germinal center reactions, and further link *GART* to immune processes associated with COVID-19 disease severity.

## Conclusions

This work implicates genetic variation in the *cis*-regulatory architecture of immune cells as a potential source of the observed variation in COVID-19 disease severity. The initial GWAS implicated candidate effector genes using metrics of linear proximity to the GWAS signal and public expression quantitative trait loci (eQTLs) datasets [25]. Several of our V2G attributions agreed with these analyses, including *OAS1*, *OAS2*, and *OAS3* as the likely effector genes for rs10774671. *DLX3*, *IL10RB*, and *IFNAR2* were also implicated in the prior study *via* intersection with eQTLs from various tissues and cell types. However, a number of GWAS candidate genes were not implicated in our study, and our immune-focused PCC maps identified several candidate effectors not implicated previously: *IFNAR1*, *GART*, S*ON*, and *AP00295.9* for rs1305728 and *TNFAIP8L1* and *FEM1A* for rs77534576. In addition, our V2G mapping of genes involved in COVID severity identified *GART* as a novel target whose activity could potentially be promoted for better anti-viral humoral immune responses or inhibited as a potential treatment for systemic autoimmune disease. Further work is necessary to determine the roles of these genes in COVID-19, and whether genes implicated here may represent therapeutic targets for COVID-19 and other inflammatory disorders.

## Methods

### Antibodies

Anti-human CD19-APC-Cy7 (HIB19, cat#302218), CD27 PerCP-Cy5.5 (O323, cat#302820), and IgD-Pacific-Blue (IA6-2, cat#348225) were from Biolegend. Anti-human CD38-APC (HIT2, cat#555462) and CD21 Pe B-ly4 (557327) were from BD Biosciences.

### Purification of B cells from human tonsil

Fresh tonsils were obtained as discarded surgical waste from de-identified immune-competent children undergoing tonsillectomy to address airway obstruction or a history of recurrent tonsillitis. These studies were approved by The Children’s Hospital of Philadelphia Institutional Review Board as non-human subject research. The mean age of donors was 5.6 years (range 3-16 years) and 75% were male. Tonsillar mononuclear cells were isolated from tissues by mechanical disruption (tonsils were minced and pressed through a 70-micron cell screen) followed by Ficoll-Paque centrifugation. Naïve B cells (CD19^+^CD21^+^IgD^+^CD38^−^) and GC B cells (CD19^+^CD21^+^CD38^+^IgD^−^CD27^−^) were then sorted using a MoFlo Astrios EQ (Beckman Coulter). The gating strategy is shown in Additional file 1 Fig S6.

### Tonsillar organoid preparation and staining

Excised tonsils from three de-identified immunocompetent patients were diced and strained through a 100-μm filter. A single cell suspension of tonsillar mononuclear cells (MNCs) was created with Ficoll density gradient separation. Once isolated, MNC were counted and resuspended in organoid media (RPMI with L-glutamine, 10% FBS, 2 mM glutamine, 1X penicillin-streptomycin, 1 mM sodium pyruvate, 1X MEM non-essential amino acids, 10 mM HEPES buffer and 1 μg/ml of recombinant human B cell activating factor [BioLegend]) at a concentration of 6 × 10^7^ cells per ml. As previously described by Wagar et al. [32], MNC’s were transferred to permeable transwells (0.4-μm pore, 12-mm diameter; Millipore). Transwells were inserted into standard 12-well polystyrene plates containing 1 ml of additional organoid media and placed in an incubator at 37 °C and 5% CO2. Lometrexol in phosphate-buffered saline was added, or not, to organoids after 48 h in culture. Organoid media with or without lometrexol was replaced every 3 days. On culture day 7, organoid MNCs were resuspended and stained at 4 °C with the anti-human CD38 (HIT2; BioLegend), CD27 (O323; BioLegend), CD19 (HIB19; BioLegend), and L/D aqua (Invitrogen). Cells were analyzed with LSRFortessa (BD Bioscience) and visualized with FlowJo software (TreeStar).

### Cell fixation

We used standard methods for cell fixation [26, 28–31]. Briefly, 10^7^ naïve or germinal center B cells were suspended in 10 mL RPMI + 10% FBS, followed by an additional 270μL of 37% formaldehyde and incubation for 10 min at RT on a platform rocker. The fixation reaction was quenched by the addition of 1.5 mL cold 1 M glycine (4 °C). Fixed cells were centrifuged at 210×*g* for 5 min at 4 °C and supernatants were removed. The cell pellets were washed in 10-ml cold PBS (4 °C) followed by centrifugation as above. Cell pellets were resuspended in 5-ml cold lysis buffer (10 mM Tris pH 8, 10 mM NaCl, 0.2% NP-40/Igepal supplemented with a protease inhibitor cocktail). Resuspended cell pellets were incubated for 20 min on ice, centrifuged at 680×*g*, and lysis buffer was removed. Cell pellets were resuspended in 1 mL of fresh lysis buffer, transferred to 1.5-mL Eppendorf tubes, and snap frozen in ethanol/dry ice or liquid nitrogen. Frozen cell pellets were stored at − 80 °C for 3C library generation.

### 3C library generation

We used standard methods for generation of 3C libraries [26, 28–31]. For each library, 10^7^ fixed cells were thawed at 37 °C, followed by centrifugation at RT for 5 min at 1845×*g*. The cell pellet was resuspended in 1 mL of dH2O supplemented with 5 μL 200X protease inhibitor cocktail, incubated on ice for 10 min, then centrifuged. Cell pellet was resuspended to a total volume of 650 μL in dH2O. Fifty microliters of cell suspension was set aside for pre-digestion QC, and the remaining sample was divided into 3 tubes. Both pre-digestion controls and samples underwent a pre-digestion incubation in a Thermomixer (BenchMark) with the addition of 0.3%SDS, 1x NEB DpnII restriction buffer, and dH2O for 1 h at 37 °C shaking at 1000 rpm. A 1.7% solution of Triton X-100 was added to each tube and shaking was continued for another hour. After pre-digestion incubation, 10 μL of DpnII (NEB, 50 U/μL) was added to each sample tube only and continued shaking along with pre-digestion control until the end of the day. An additional 10 μL of DpnII was added to each digestion reaction and digested overnight. The next day, a further 10 μL DpnII was added and continue shaking for another 2–3 h. One hundred microliters of each digestion reaction was then removed, pooled into one 1.5-mL tube, and set aside for digestion efficiency QC. The remaining samples were heat inactivated incubated at 1000 rpm in a MultiTherm for 20 min at 65 °C to inactivate the DpnII and cooled on ice for 20 additional minutes. Digested samples were ligated with 8 μL of T4 DNA ligase (HC ThermoFisher, 30 U/μL) and 1X ligase buffer at 1000 rpm overnight at 16 °C in a MultiTherm. The next day, an additional 2 μL of T4 DNA ligase was spiked into each sample and incubated for another few hours. The ligated samples were then de-crosslinked overnight at 65 °C with Proteinase K (20 mg/mL, Denville Scientific) along with pre-digestion and digestion control. The following morning, both controls and ligated samples were incubated for 30 min at 37 °C with RNase A (Millipore), followed by phenol/chloroform extraction, ethanol precipitation at − 20 °C, the 3C libraries were centrifuged at 1000×*g* for 45 min at 4 °C to pellet the samples. The controls were centrifuged at 1845×*g*. The pellets were resuspended in 70% ethanol and centrifuged as described above. The pellets of 3C libraries and controls were resuspended in 300 μL and 20 μL dH2O, respectively, and stored at − 20 °C. Sample concentrations were measured by Qubit. Digestion and ligation efficiencies were assessed by gel electrophoresis on a 0.9% agarose gel and also by quantitative PCR (SYBR green, Thermo Fisher).

### Promoter-Capture-C design

Our promoter-Capture-C approach was designed to leverage the four-cutter restriction enzyme *DpnII* in order to give high resolution restriction fragments of a median of ~250bp [26, 28–31]. This approach also allows for scalable resolution through in silico fragment concatenation. Custom capture baits were designed using Agilent SureSelect RNA probes targeting both ends of the *DpnII* restriction fragments containing promoters for coding mRNA, non-coding RNA, antisense RNA, snRNA, miRNA, snoRNA, and lincRNA transcripts (UCSC lincRNA transcripts and sno/miRNA under GRCh37/hg19 assembly) totaling 36,691 RNA baited fragments through the genome. The capture library was re-annotated under gencodeV19 at both 1-fragment and 4-fragment resolution.

### Promoter-Capture-C assay

Isolated DNA from 3C libraries was quantified using a Qubit fluorometer (Life technologies), and 10 μg of each library was sheared in dH2O using a QSonica Q800R to an average fragment size of 350bp [26, 28–31]. QSonica settings used were 60% amplitude, 30s on, 30s off, 2-min intervals, for a total of 5 intervals at 4 °C. After shearing, DNA was purified using AMPureXP beads (Agencourt). DNA size was assessed on a Bioanalyzer 2100 using a DNA 1000 Chip (Agilent) and DNA concentration was checked via Qubit. SureSelect XT library prep kits (Agilent) were used to repair DNA ends and for adaptor ligation following the manufacturer’s protocol. Excess adaptors were removed using AMPureXP beads. Size and concentration were checked by Bioanalyzer using a DNA 1000 Chip and by Qubit fluorometer before hybridization. One microgram of adaptor-ligated library was used as input for the SureSelect XT capture kit using manufacturer protocol and our custom-designed 41 K promoter Capture-C library. The quantity and quality of the captured library was assessed by Bioanalyzer using a high sensitivity DNA Chip and by Qubit fluorometer. SureSelect XT libraries were then paired-end sequenced on 8 lanes of Illumina Hiseq 4000 platform (100 bp read length).

### ATAC-seq library generation

The tonsillar T cells [26], monocytes [69], and B cell subsets were processed in the same manner. A total of 50,000 to 100,000 sorted cells were centrifuged at 550 *g* for 5 min at 4 °C. The cell pellet was washed with cold PBS and resuspended in 50 μL cold lysis buffer (10 mM Tris-HCl, pH 7.4, 10 mM NaCl, 3 mM MgCl2, 0.1% NP-40/IGEPAL CA-630) and immediately centrifuged at 550 *g* for 10 min at 4 °C. Nuclei were resuspended in the Nextera transposition reaction mix (25 μL 2x TD Buffer, 2.5 μL Nextera Tn5 transposase (Illumina Cat #FC-121-1030), and 22.5 μL nuclease free H_2_O) on ice, then incubated for 45 min at 37 °C. The tagmented DNA was then purified using the Qiagen MinElute kit eluted with 10.5 μL Elution Buffer (EB). Ten microliters of purified tagmented DNA was PCR amplified using Nextera primers for 12 cycles to generate each library. PCR reaction was subsequently cleaned up using 1.5x AMPureXP beads (Agencourt), and concentrations were measured by Qubit. Libraries were paired-end sequenced on the Illumina HiSeq 4000 platform (100 bp read length).

### ATAC-seq analysis

ATAC-seq peaks from libraries of tonsillar T cells [26], monocytes [69], and B cell subsets were called using the ENCODE ATAC-seq pipeline (https://www.encodeproject.org/atac-seq/). Briefly, pair-end reads from three biological replicates for each cell type were aligned to hg19 genome using bowtie2, and duplicate reads were removed from the alignment. Narrow peaks were called independently for each replicate using macs2 (-p 0.01 --nomodel --shift -75 --extsize 150 -B --SPMR --keep-dup all --call-summits) and ENCODE blacklist regions (ENCSR636HFF) were removed from peaks in individual replicates. The IDR optimal peak set for each cell type was used to define open chromatin regions in this study.

### Promoter-focused Capture-C analysis

Paired-end reads from three biological replicates were pre-processed using the HiCUP pipeline (v0.5.9) [70], with bowtie2 as aligner and hg19 as the reference genome. Significant promoter interactions at 1-DpnII fragment resolution were called using CHiCAGO (v1.1.8) [71] with default parameters except for binsize set to 2500. Significant interactions at 4-DpnII fragment resolution were also called using CHiCAGO with artificial .baitmap and .rmap files in which DpnII fragments were concatenated in silico into 4 consecutive fragments using default parameters except for removeAdjacent set to False. The significant interactions (CHiCAGO score > 5) from both 1-fragment and 4-fragment resolutions were exported in .ibed format and merged into a single file using custom a PERL script to remove redundant interactions and to keep the max CHiCAGO score for each interaction.

### COVID-19 GWAS data integration

We curated the lead SNPs from the recent COVID-19 severity GWAS from the COVID-19 Host Genetics Initiative [25] (Freeze 5). The six genome-wide significant SNPs associated with critical illness and COVID-19 severity were selected for investigation in our study. We identified proxies in LD with significant COVID-19 severity loci using LDLinkR with *R*^2^ > 0.8 in EUR ancestry. We next intersected the COVID-19 sentinel and proxy SNPs with the set of OCR annotated to promoter regions (−1500/+ 500 bp of TSS) and OCR overlapping promoter interacting regions identified by Capture C. Genomic coordinate overlaps were identified using the R package GenomicRanges (ver 1.42) against the human genome reference hg19.

### Partitioned heritability LD score regression enrichment analysis

Partitioned heritability LD score regression (v1.0.0) was used to identify enrichment of GWAS summary statistics among open accessible regions identified in each cell type. The baseline analysis was performed using LDSCORE data (https://data.broadinstitute.org/alkesgroup/LDSCORE) with LD scores, regression weights, and allele frequencies from 1000G Phase1 and summary statistics from the COVID-19 Host Genetics Initiative [25]. We generated partitioned LD score regression annotations for each cell type using the coordinates of the all promoter OCR + promoter-interacting OCR. Finally, the cell-type-specific partitioned LD scores were compared to baseline LD scores to measure enrichment in each cell type independently.

### Transcription factor motif analysis

Transcription factor binding site motifs overlapping with proxies implicated in by variant to gene mapping analysis were identified using the R package motifbreakR (v2.0.0) [72] using the Jaspar2018 database as our reference set of position weight matrices [73]. Results were filtered to TFs that expressed in the implicated cell type (TPM > 1) and were visualized using Cytoscape (v3.8.2) [74].

### RNA-seq library generation and analysis

RNA was isolated from ~ 1 million of each cell type using Trizol Re- agent (Invitrogen), purified using the Directzol RNA Miniprep Kit (Zymo Research), and depleted of contaminating genomic DNA using DNAse I. Purified RNA was checked for quality on a Bioanlayzer 2100 using the Nano RNA Chip and samples with RIN > 7 were used for RNA-seq library preparation. RNA samples were depleted of rRNA using QIAseq Fastselect RNA removal kit (Qiagen). Samples were then processed for the preparation of libraries using the SMARTer Stranded Total RNA Sample Prep Kit (Takara Bio USA) according to the manufacturer’s instructions. Briefly, the purified first-strand cDNA is amplified into RNA-seq libraries using SeqAmp DNA Polymerase and the Forward and the Reverse PCR Primers from the Illumina Indexing Primer Set HT for Illumina. Quality and quantity of the libraries was assessed using the Agilent 2100 Bioanalyzer system and Qubit fluorometer (Life Technologies). Sequencing of the finalized libraries was performed on the NovaSeq 6000 platform at the CHOP Center for Spatial and Functional Genomics. For analysis, TPM values of bulk RNA-seq data were calculated using the pseudo-aligner Kallisto 0.46.2 [75]. Gene level expression was calculated by combining individual TPM values. The comparison of gene expression level between those with promoters with contacts to OCRs or those lacking contacts with OCRs was performed using an unpaired two-sided Wilcoxon rank-sum test, implemented in the R function wilcox.test.

### COVID-19 V2G gene integration with external RNA-seq datasets

We retrieved bulk RNA-seq fastq datasets associated with Galani et al. [33] from the Sequence Read Archive using sra-tools (SRA Toolkit Development Team; http://ncbi.github.io/sra-tools/) and plotted the log2 fold change data reported for severe or mild COVID-19 compared to the mean of 5 healthy donors. Single-end fastq files were retrieved with fastq-dump. TPM values were calculated using kallisto with the following parameters: --single -l 200 -s 20. The log_2_FC for the genes implicated in our immune cell types was calculated for each COVID19 patient compared. We retrieved processed data for pseudo-bulk comparisons between severe and mild COVID-19 relative to healthy donors from Zhang et al. [34].

### Ingenuity pathway analysis

Ingenuity pathway analysis (IPA, QIAGEN) was used for gene ontology analysis.

## Supplementary Information

**Additional file 1: Figures S1-S6 and Supplemental Table Legends.** **Figure S1.** Summary of ATAC-seq datasets. **Figure S2.** Summary of Capture C datasets. **Figure S3.** Genomic Tracks indicating the position of SNPs of interest. **Figure S4.** Top IPA gene ontology network for genes implicated by COVID-19 V2G. **Figure S5.** The top IPA gene ontology network for transcription factors whose binding is predicted to be influenced by COVID-19-associated SNPs. **Figure S6.** Gating strategy for sorting of naïve B cells and GCB cells for this study.

**Additional file 2: Table S1** – *Summary of ATAC-seq peak calls.*

**Additional file 3: Table S2** – *Summary of interactions called in PCC*.

**Additional file 4: Table S3** – *COVID-19 variant-to-gene mapping (V2G)* using Capture C and ATAC-seq.

**Additional file 5: Table S4** - *COVID-19 V2G gene set IPA analysis*.

**Additional file 6: Table S5** – *Modeling COVID-19-associated SNP effects on TF*.

**Additional file 7: Table S6** – *COVID-19 SNP-effected TF gene set IPA analysis.*

**Additional file 8: **Review history – *Peer review history*.

## Data Availability

Monocyte and naïve and germinal center B cell raw ATAC seq and Capture C datasets are deposited in GEO with the accession number GSE174658 [76]. The naïve CD4+ T cell and TFH datasets are available at ArrayExpress (https://www.ebi.ac.uk/arrayexpress/) with accession numbers E-MTAB-6621 (promoter-Capture-C) and E-MTAB-6617 (ATAC-seq) [77]. COVID-19 severity-associated sentinel SNPs were retrieved from Supplemental Table 2 of the COVID-19 Host Genetics Initiative. COVID19 summary stats were retrieved from Freeze 5 of COVID19 Host initiative (https://www.covid19hg.org/results/r5/) [78]. Gene expression data for severe COVID19 was retrieved from the SRA from BioProject PRJNA638753 [79].
