# Supplementary material for: Implicating effector genes at COVID-19 GWAS loci using promoter-focused Capture-C in disease-relevant immune cell types
Source: Genome Biol. 2022 Jun 3;23:125. doi: 10.1186/s13059-022-02691-1 (PMC9164584; doi:10.1186/s13059-022-02691-1)
Supplement: Supplementary file 1 — Additional file 1: Figures S1-S6 and Supplemental Table Legends. Figure S1. Summary of ATAC-seq datasets. Figure S2. Summary of Capture C datasets. Figure S3. Genomic Tracks indicating the position of SNPs of interest. Figure S4. Top IPA gene ontology network for genes implicated by COVID-19 V2G. Figure S5. The top IPA gene ontology network for transcription factors whose binding is predicted to be influenced by COVID-19-associated SNPs. Figure S6. Gating strategy for sorting of naïve B cells and GCB cells for this study. [file 13059_2022_2691_MOESM1_ESM.pdf]

**Figure S1**

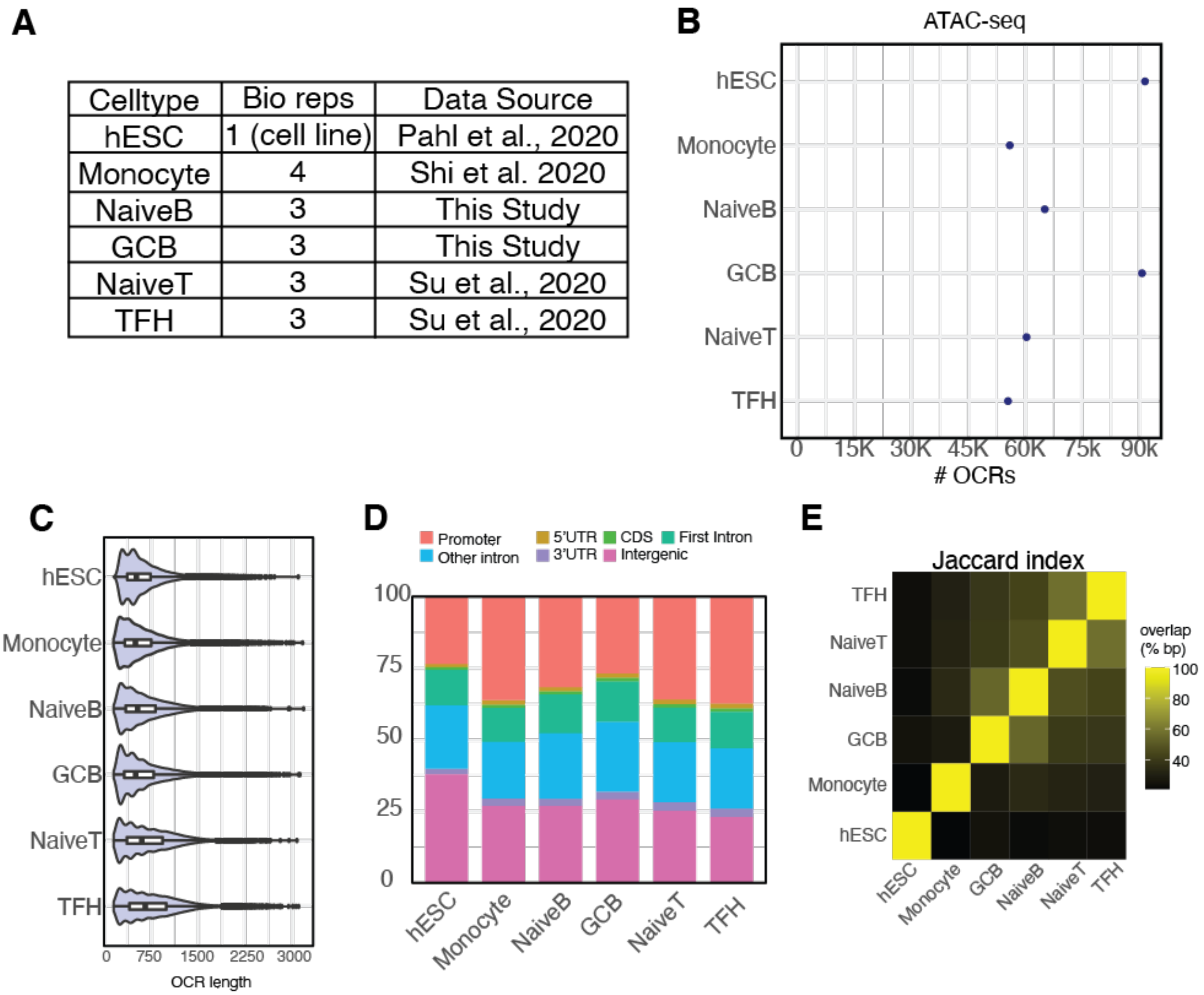

| Celltype | Bio reps | Data Source |
|---|---|---|
| hESC | 1 (cell line) | Pahl et al., 2020 |
| Monocyte | 4 | Shi et al. 2020 |
| NaiveB | 3 | This Study |
| GCB | 3 | This Study |
| NaiveT | 3 | Su et al., 2020 |
| TFH | 3 | Su et al., 2020 |



Source of ATAC-seq datasets for hESCs, monocytes, naïve B cells, GCB, naïve CD4+ T cells, and TFH. (A) The number of biological replicates coming from distinct genetic backgrounds and the source publication for each of the datasets used in this study. hESC data was derived from the H09 cell line. (B) Number of open chromatin regions (ENCODE optimal IDR peaks) identified per cell type. (C) Distribution of open chromatin region lengths. (D) Annotation of genomic features overlapping with OCRs. (E) Pairwise comparison of the global open chromatin regions called as OCRs between each cell type. Yellow indicates the overlap (intersection regions (bp)/union of regions (bp) between each cell type comparison).

**Figure S2**

A

| Celltype | Bio reps | Data Source |
|---|---|---|
| hESC | 1 (cell line) | Pahl et al., 2020 |
| Monocyte | 4 | Shi et al. 2020 |
| NaiveB | 3 | This Study |
| GCB | 3 | This Study |
| NaiveT | 3 | Su et al., 2020 |
| TFH | 3 | Su et al., 2020 |

B

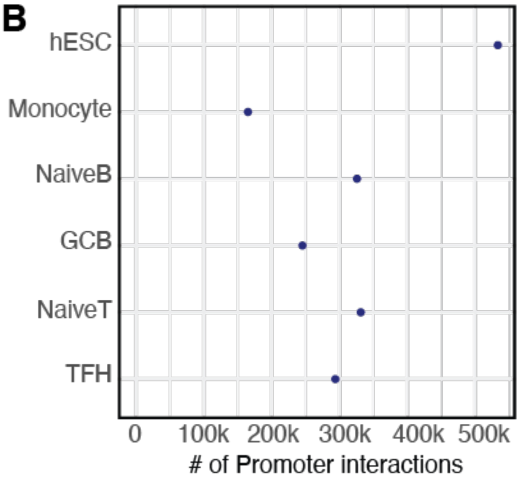


C

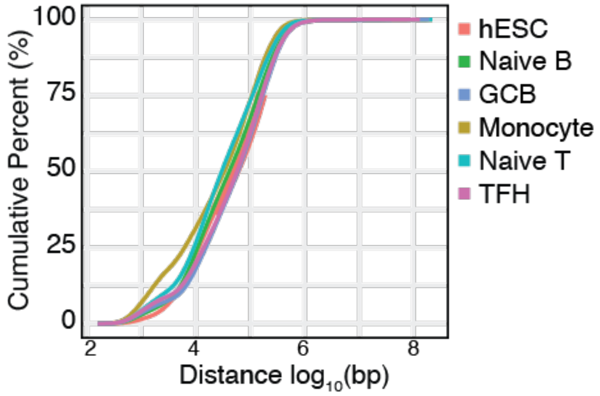


D

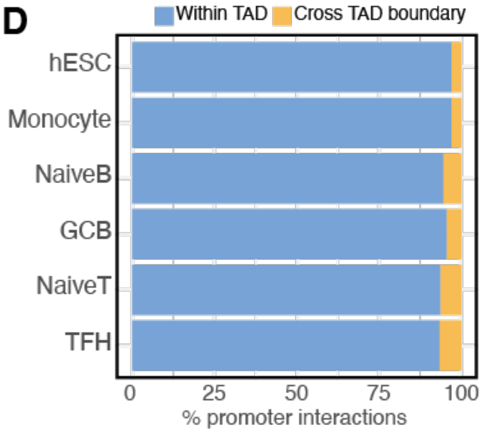


E

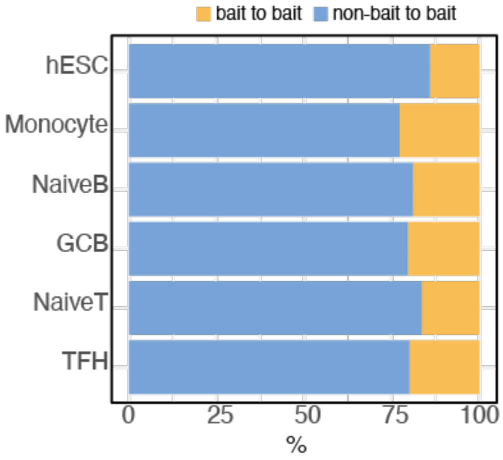


Source of Capture C data for hESCs, monocytes, naïve B cells, GCB, naïve CD4+ T cells, and TFH. (A) The number of biological replicates coming from distinct genetic backgrounds and the source publication for each of the datasets used in this study. (B) Number regions identified to interact with a promoter. (C) Cumulative distribution of distances between baits and interacting regions. (D) Proportion of promoter interaction that are located within TADs or that cross TADs. (E) The proportion of promoter interactions that were called in bait-to-bait interactions.

**Figure S3**

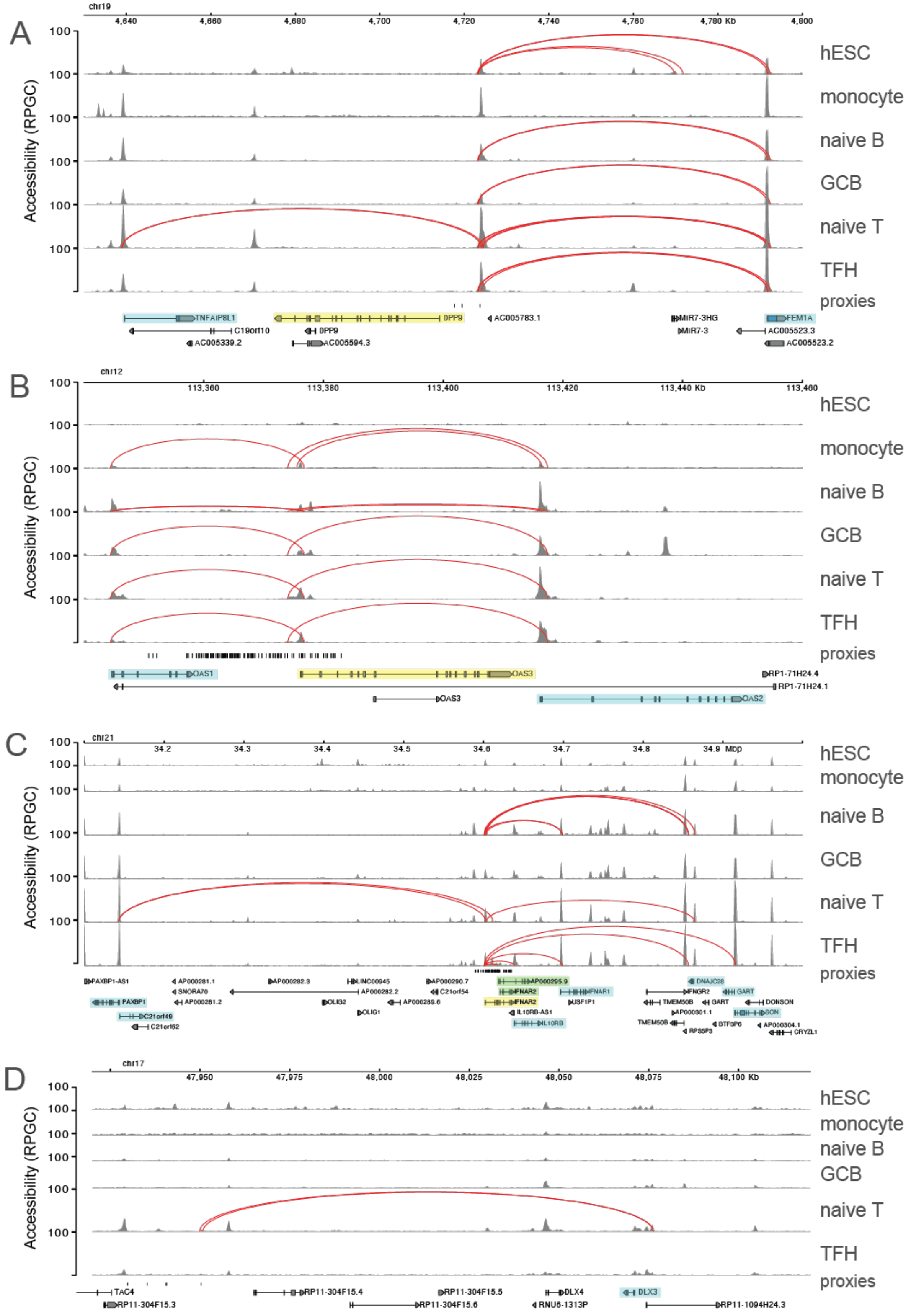

UCSC browser tracks depicting chromatin accessibility (grey), promoter interactions (red) and proxy SNPs (black) at each COVID-19 GWAS locus in each cell type.

**Figure S4**

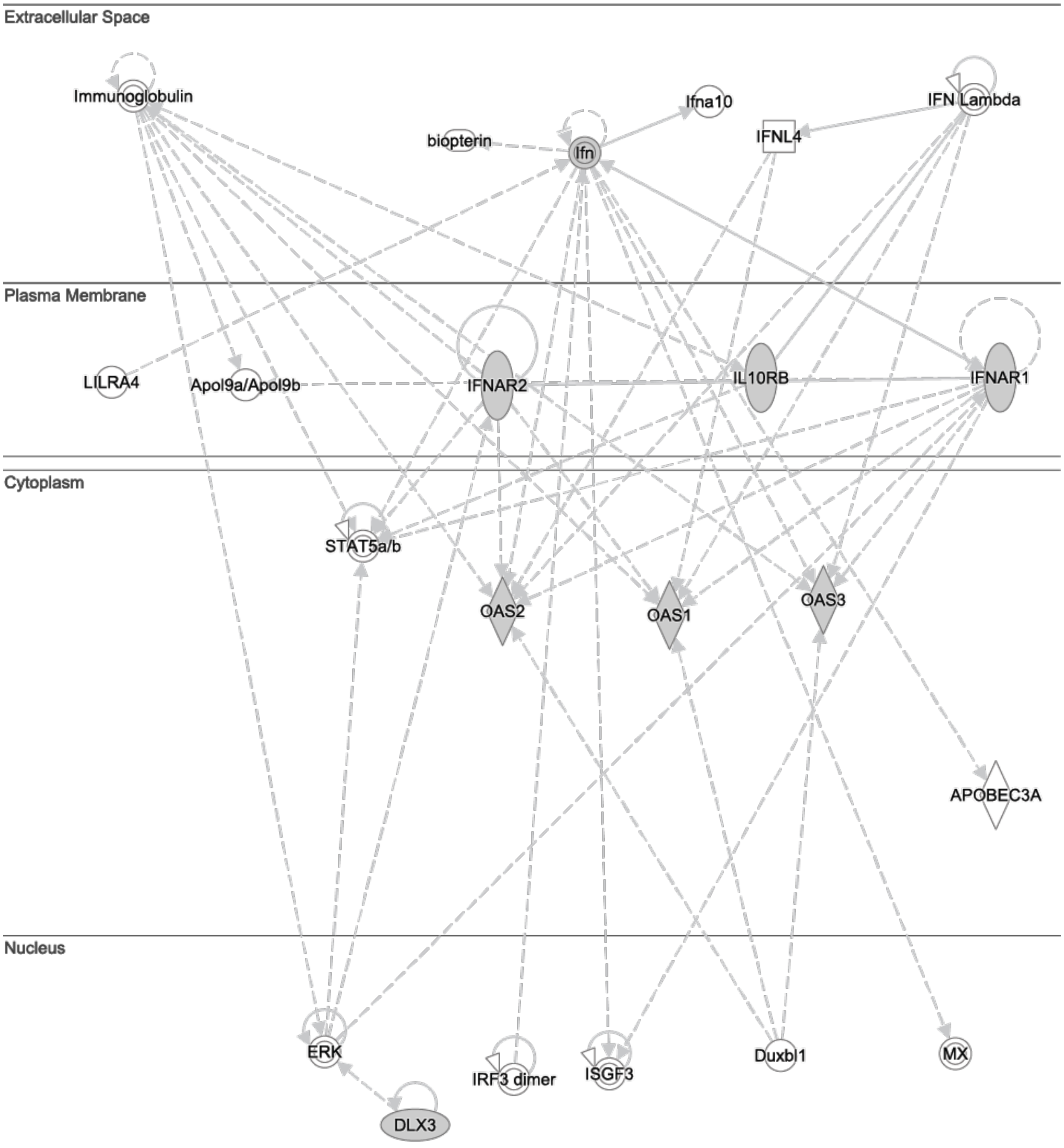


Top IPA gene ontology network for genes implicated by COVID-19 V2G.

**Figure S5**

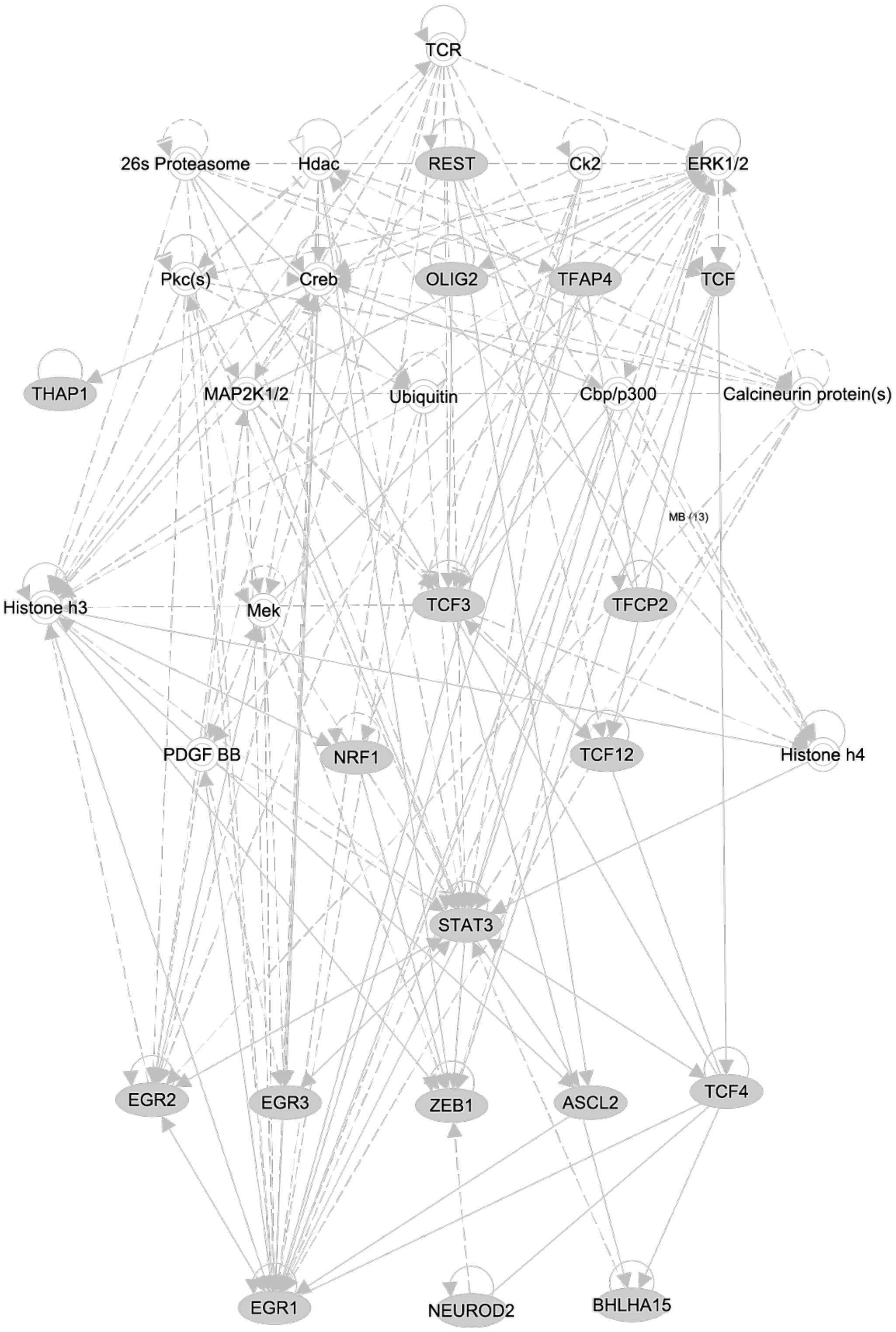


The top IPA gene ontology network for transcription factors whose binding is predicted to be influenced by COVID-19-associated SNPs.

**Figure S6**

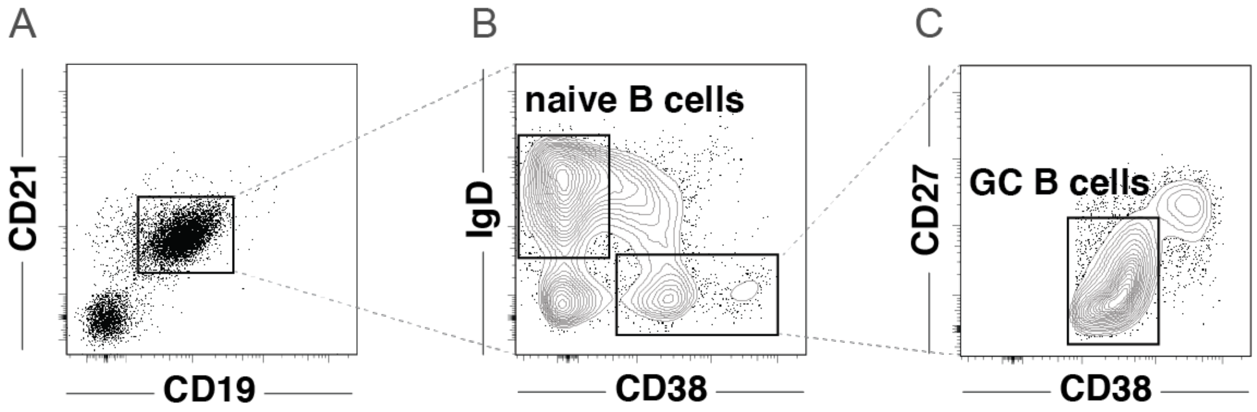


Gating strategy for sorting of naïve B cells and GCB cells for this study.

**Supplementary Table Legends**

**Table S1** – *Summary of ATAC-seq peak calls* - The number and median length from the IDR optimal peak set called by the ENCODE pipeline. The percent of OCRs annotated to different genomic regions (Promoters, 5'UTRs, CDS, First introns, other introns, 3'UTRs, and intergenetic regions.

**Table S2** – *Summary of interactions called in PCC* - The number and median distance between interacting fragments. The percentage of loops within annotated TAD boundaries. Percent of interactions called between two bait fragments. Number of OCRs contacted or not contacted by a promoter interaction, and the percentage of OCRs with promoter contacts.

**Table S3 –** *COVID-19 variant-to-gene mapping (V2G)* - The proxies queried for each sentinel with the corresponding proxy coordinates (hg19) $R^2$ value. The proxyOCR indicates the location of the proxy SNP. Cell indicates in which cell type the variant to gene call was made, proxyOCR indicates the genomic coordinates of the OCR that contains the proxy. Gene ID and gene name of the implicated genes. Frag indicates the resolution the interaction was called, b2b: whether the interaction was called in a bait to bait interaction. Score indicates the Chicago score (-log adjusted P value). Proxy2BaitDistance is the distance between the proxy and the bait. Other end frag and bait frag are the coordinates of the two ends of the interacting regions. If the interaction was a bait to bait call, oeGenes indicates which gene the bait to bait is annotated. For proxies in promoters, proxy2GeneDist indicates the distance between the proxy and TSS of the indicated gene. SNPs located in promoters have NAs for Capture C information.

**Table S4 -** *COVID-19 V2G gene set IPA analysis* – IPA analysis for the set of all genes implicated in the V2G mapping: Canonical pathways, Upstream regulators, Causal Networks, Diseases and Biological Functions. Enrichment *P* values were calculated from Fisher's Exact test.

**Table S5 –** *Modeling COVID-19-associated SNP effects on TF binding* – Disruption of TF binding by V2G-implicated proxies was predicted using motifBreakR with position weight matrices queried from JASPAR2018. For each PCC-established SNP-Gene pair, the motif score (scoreRef/scoreAlt) indicates TF binding site score for both reference and alternative alleles along with the associated *P* value (Refpvlaue/Altpvalue). Effect indicates whether the swap is expected to have a weak or strong effect on motif binding. TF_gene_name/TF_gene_id: TF binding site predicted to be affected by the proxy. TF_tpm is the mean TPM value for the TF in the given cell type.

**Table S6 –** *COVID-19 SNP-effected TF gene set IPA analysis* – IPA analysis for the set of transcription factors with binding sites predicted to be disrupted by V2G implicated proxy SNPs: Canonical pathways, Upstream regulators, Causal Networks, Diseases and Biological Functions. Enrichment *P* values were calculated from Fisher's Exact test.
