# Supplementary material for: Implicating effector genes at COVID-19 GWAS loci using promoter-focused Capture-C in disease-relevant immune cell types
Source: Genome Biol. 2022 Jun 3;23:125. doi: 10.1186/s13059-022-02691-1 (PMC9164584; doi:10.1186/s13059-022-02691-1)
Supplement: Supplementary file 8 — Additional file 8: Review history – Peer review history. [file 13059_2022_2691_MOESM8_ESM.docx]

**1^st^ round**

**Reviewer 1**

Pahl et al. have conducted an interesting study to leverage ATAC-seq and high-resolution promoter-focused Capture-C (PCC) techniques in primary naive B cells, naïve CD4+ T cells, follicular helper T cells and germinal center B cells from human tonsil, and circulating monocytes to gain insights into genes implicated in COVID-19 severity. While this manuscript is in general clearly written and is an interesting addition to already existing literature of putative effector genes associated with COVID-19 severity, I have some further questions and comments.

Major comments:

1. The section about immune dynamics of SARS-CoV-2 infection is concluded by highlighting CD4+ T cells, TFH, and germinal center B cells (GCB) as the likely influencers of the severity of COVID-19 disease. As a conclusion of results presented on Figure 1, CD4+ T cell and monocyte are highlighted, which is claimed to be consistent with the immune dynamics observed in patients. I may seem to miss the basis of this claim - why are monocytes more important than GCBs based on the section of immune dynamics of SARS-CoV-2 infection?

2. COVID-19 GWAS data - was the COVID-19 GWAS data by the COVID-19 HGI or Pairo-Castineira et al.? The first one is mentioned in the Methods section, but the second one is referenced in the legend of Figure 2A. Please also note that COVID-19 HGI (freeze 5) identified 13 genome-wide significant loci that are associated with SARS-CoV-2 infection or severe manifestations of COVID-19, but you seem to focus only on COVID-19 severity variants (line 350-352). How many COVID-19 severity loci did you investigate? Please highlight this also in the main text. What was the LD threshold used for identifying proxies? Both r2 > 0.8 and r2 >0.6 are highlighted in the methods section. Please highlight this also in the main text.

3. COVID-19 GWAS data on Figure 2 - according to the legend of Figure 2 genome-wide significant signals are highlighted but based on the figure it can be seen that some genome-wide significant signals are missed. Please clarify. As a conclusion based on the results, it said that "This study implicated multiple genes at all but one locus" (lines 161-162), but based on Figure 2B only 4 out 6 loci are further discussed. Please clarify.

4. The authors identified 16 genes whose promoters physically interact with accessible COVID-19-associated variants in immune cells - do I understand correctly that this could mean that there is an accessible genetic variant (termed "open proxy" or "accessible proxy"? - what's the difference?) in the promoter region of a given gene in LD with the GWAS variant? And accessibility of a genetic variant is defined based on ATAC-sec? Should one check if the open proxy has a regulatory effect on the gene to claim pleiotropic effects of COVID-19 disease risk-associated genetic variants?

5. Comparison between COVID-19 patients and healthy controls - this analysis needs more details. What statistical test is used to compare the expression of genes from COVID-19 patients to healthy donors? How is FDR calculated? Was there information available for relevant confounders, such as age, sex, etc to account for in the analysis? What do P15, P22, etc, represent on Figure 3A? How was comparison between severe vs mild COVID-19 patients done? Also, I find it confusing that Figure 3A and 3B (COVID-19 patients vs healthy donors) are used to illustrate differences between sever and mild COVID patients (lines 131-137). Should fold change be relative to either sever or mild disease for a more meaningful illustration?

Minor comments:

1. Were Supplementary Tables uploaded with the submission? I seem to miss these.

2. Typo: Wilcoxian rank-sum test -> Wilcoxon rank-sum test

3. Figure 1B: Were there no outlying data points? Also, could you give some statistics about the genes with promoter-OCR contacts, e.g. range? For clarity should the labels include "promoter", such as "promoter-connected OCR gene" instead of just "OCR-connected gene"?

4. Please add color legend for Figure 2B.

5. Lines 106-107: "The 'DPP9' sentinel rs2109069 …" - rs2109069 is an intronic variant in the DPP9 gene. I find it confusing to use quotes here. What should it represent?

6. Figure 2C - should "naiveT" be "naive CD4" as in Figure 1C? Please be consistent.

7. Lines 110-111: "The sentinel rs13050728 is in LD with open proxies interacting with … IFNAR1 and DNAJC28 in naive T and B cells …". Based on the Figure 2C, IFNAR1 is highlighted in TFH and naïve B cells.

8. Figure 3 - please highlight significant differences on the plot. For example, an asterisk (*) could be added to the cell with FDR < 0.05.

9. Line 131-132: "Importantly, six of these genes also exhibited severity-associated patterns of expression" - in total of five genes are highlighted on Figure 3A and Figure 3B.

10. The gating strategy is shown in Supplementary Figure 6 instead of Supplementary Figure 7 (lines 233-234).

**Reviewer 2**

This is very solid work, with high quality and valuable data generated on whole genome ATAC-seq and Capture C on a number of primary cell lines. This data is then used for a targetted analysis of a particular GWAS associated region.

The analysis of the SARs-Cov19 associated regions is also solid, with appropriate analysis of the independent signals and proxy SNPs. There is also relevant and accurate bioinformatic analysis performed from public data sources.

The work is presented very clearly and is very well written.

I think to demonstrate a clear advance from the GWAS signals to putative causal genes in specific cell types, over and above publicly available data on expression, TFBS and gene ontology and the in-house whole genome generated data on activity/interactions, at least one of the interactions needs to be validated as being functional in a particular cell type.

**Authors’ response**

Reviewer 1 - Pahl et al. have conducted an interesting study to leverage ATAC-seq and high-resolution promoter-focused Capture-C (PCC) techniques in primary naive B cells, naïve CD4+ T cells, follicular helper T cells and germinal center B cells from human tonsil and circulating monocytes to gain insights into genes implicated in COVID-19 severity. While this manuscript is in general clearly written and is an interesting addition to already existing literature of putative effector genes associated with COVID-19 severity.

RESPONSE - We thank the reviewer for their appreciation of the value of our work.

Reviewer 1 - The section about immune dynamics of SARS-CoV-2 infection is concluded by highlighting CD4+ T cells, TFH, and germinal center B cells (GCB) as the likely influencers of the severity of COVID-19 disease. As a conclusion of results presented on Figure 1, CD4+ T cell and monocyte are highlighted, which is claimed to be consistent with the immune dynamics observed in patients. I may seem to miss the basis of this claim - why are monocytes more important than GCBs based on the section of immune dynamics of SARS-CoV-2 infection?

RESPONSE – The reviewer raises an important point. Monocytes certainly are important in COVID-19, and we indicated in the introduction that monocyte-derived macrophages are a major target for SARS-CoV2 infection in the lung and a source of T cell chemoattractants during COVID-19 (see lines 54-56). Most immunologic studies of COVID-19 have particularly implicated TFH cells, antibody-producing B cells, and SARS-CoV2-specific antibodies with disease severity, as described in the introduction. Indeed, our analysis in Figure 1C shows enrichment of COVID-19 severity heritability in monocyte, TFH and naïve CD4+ T cell open chromatin, with naïve CD4+ T cell heritability reaching FDR<0.05. This suggests that monocytes and CD4+ T cells are cell types whose function is most likely impacted by polymorphisms associated with COVID-19 severity. While GCB cell open chromatin is not enriched for COVID-19 severity heritability, we do not conclude that GCB cells are not important to COVID immunity in general. To clarify this issue, we have revised lines 100-102 to read:

“These results are consistent with the growing evidence that multiple immune cell types contribute to disease severity and suggests that COVID-19-associated variants in CD4+ T cell and monocyte open chromatin may have the strongest influence on disease severity.”

Reviewer 1 - COVID-19 GWAS data - was the COVID-19 GWAS data by the COVID- 19 HGI or Pairo-Castineira et al.? The first one is mentioned in the Methods section, but the second one is referenced in the legend of Figure 2A. Please also note that COVID-19 HGI (freeze 5) identified 13 genome-wide significant loci that are associated with SARS-CoV-2 infection or severe manifestations of COVID-19, but you seem to focus only on COVID-19 severity variants (line 350-352). How many COVID-19 severity loci did you investigate? Please highlight this also in the main text. What was the LD threshold used for identifying proxies? Both r2>0.8 and r2>0.6 are highlighted in the methods section. Please highlight this also in the main text.

RESPONSE - We thank the reviewer for pointing out this error. While preparing our manuscript, the updated COVID-19 HGI GWAS was published, so we subsequently updated our analyses to integrate these most recent GWAS data. Likewise, only the r2>0.8 cutoff was used in our current version of the manuscript, and although an r2>0.6 threshold implicated the same genes, we have removed the reference to r2>0.6 in the methods section. We have added this information to lines 105-109 of the manuscript: “To map potentially functional COVID-19 variants to their target genes, we intersected our ATAC-seq and PCC data with the most recent COVID-19 disease risk GWAS25 (Figure 2 A and B, Supplementary Table 3). We examined proxies in high LD (r2>0.8) of the six independent genome wide significant signals associated with COVID19 severity and identified 16 genes whose promoters physically interact with accessible COVID-19-associated variants in immune cells”

Reviewer 1 - COVID-19 GWAS data on Figure 2 - according to the legend of Figure 2 genome-wide significant signals are highlighted but based on the figure it can be seen that some genome-wide significant signals are missed. Please clarify. As a conclusion based on the results, it said that "This study implicated multiple genes at all but one locus" (lines 161-162), but based on Figure 2B only 4 out 6 loci are further discussed. Please clarify.

RESPONSE - Several of the genome-wide significant signals were not implicated from our analyses. This is likely due to these signals acting through other cellular state or contexts besides the immune cell types we assayed, such as the lung or other innate immune cell types. In fact, rs10490770 has been implicated in the regulation of lung epithelial cell function (Downes, 2022). We have now highlighted the signals where we failed to detect signal on lines 142-146.

Reviewer 1 - The authors identified 16 genes whose promoters physically interact with accessible COVID-19-associated variants in immune cells - do I understand correctly that this could mean that there is an accessible genetic variant (termed "open proxy" or "accessible proxy"? - what's the difference?) in the promoter region of a given gene in LD with the GWAS variant? And accessibility of a genetic variant is defined based on ATAC-sec? Should one check if the open proxy has a regulatory effect on the gene to claim pleiotropic effects of COVID-19 disease risk-associated genetic variants?

RESPONSE - The reviewer is correct. The terms “open proxy” and “accessible proxy” are used interchangeably in reference to proxies located within open chromatin regions. We now use the term ‘accessible’ for consistency. In future studies we plan to directly measure the impact of COVID-19 severity-associated SNPs on target gene expression, for example in recombinant reporter studies, but we first needed to implicate the target genes in this study.

Reviewer 1 - Comparison between COVID-19 patients and healthy controls - this analysis needs more details. What statistical test is used to compare the expression of genes from COVID-19 patients to healthy donors? How is FDR calculated? Was there information available for relevant confounders, such as age, sex, etc to account for in the analysis? What do P15, P22, etc, represent on Figure 3A? How was comparison between severe vs mild COVID-19 patients done? Also, I find it confusing that Figure 3A and 3B (COVID-19 patients vs healthy donors) are used to illustrate differences between sever and mild COVID patients (lines 131-137). Should fold change be relative to either sever or mild disease for a more meaningful illustration?

RESPONSE – To clarify, the dataset presented in Figure 3 represents integration of our new 3D epigenomic data with scRNA-seq data from two published reports that related gene expression with COVID19 severity. The details of these calculations are found within Galani et al., 2021 and Zhang et al., 2021.

Reviewer 1 - Were Supplementary Tables uploaded with the submission? I seem to miss these.

RESPONSE – Supplementary Tables are uploaded to the editorial system

Reviewer 1 - Typo: Wilcoxian rank-sum test -> Wilcoxon rank-sum test

RESPONSE – This typo has been corrected

Reviewer 1 - Figure 1B: Were there no outlying data points? Also, could you give some statistics about the genes with promoter-OCR contacts, e.g. range? For clarity should the labels include "promoter", such as "promoter-connected OCR gene" instead of just "OCR-connected gene"?

RESPONSE - Outliers were removed from the graph for legibility. We have included a version of the figure with outliers. The difference in terminology stems from the fact that Figure 1A is quantifying OCR while Figure 1B is quantifying expression levels of genes.

Reviewer 1 - Please add color legend for Figure 2B.

RESPONSE - A legend was added and indicates the method that implicated the gene (accessible proxies in proximity to the promoter (promoter OCR in yellow), accessible proxies with promoter capture C loops to the gene in blue, and genes implicated by proxies falling into both categories (green).

Reviewer 1 - Lines 106-107: "The 'DPP9' sentinel rs2109069 ..." - rs2109069 is an intronic variant in the DPP9 gene. I find it confusing to use quotes here. What should it represent?

RESPONSE - GWAS signals are often arbitrarily named after the nearest gene, therefore we use quotes when describing these signal-gene designations that are commonly used to distinguish between the arbitrary naming and the actual genes we observe in our approach. However, since we have not used this convention throughout the rest of the manuscript, we eliminated the quotes when referring to the DPP9 sentinel.

Reviewer 1 - Figure 2C - should "naiveT" be "naive CD4" as in Figure 1C? Please be consistent.

RESPONSE - We have corrected the labels for consistency

Reviewer 1 - Lines 110-111: "The sentinel rs13050728 is in LD with open proxies interacting with ... IFNAR1 and DNAJC28 in naive T and B cells ...". Based on the Figure 2C, IFNAR1 is highlighted in TFH and naïve B cells.

RESPONSE - We have revised the sentence (lines 117-119) to now read: “The sentinel rs13050728 is in LD with accessible proxies interacting with PAXBP1, C21orf49, and AP000295.9 in naive CD4+ T cells, IFNAR1 in TFH and naïve B cells, DNAJC28 in naive T and B cells, GART, IL10RB, and SON in TFH cells, and IFNAR2 in all cell types (Figure 2C).”

Reviewer 1 - Figure 3 - please highlight significant differences on the plot. For example, an asterisk (*) could be added to the cell with FDR < 0.05.

RESPONSE - Genes with significant differential expression between severe and mild are highlighted in red, as indicated in the figure legend. For panel A, significance was calculated considering multiple timepoints, so an asterisk in an individual cell would not be representative.

Reviewer 1 - Line 131-132: "Importantly, six of these genes also exhibited severity- associated patterns of expression" - in total of five genes are highlighted on Figure 3A and Figure 3B.

RESPONSE – This section of the text refers to two sets of differentially expressed genes: 1) genes that are differentially expressed between infected and uninfected (see lines 151-156), and 2) genes that are differentially expressed between patients with mild vs. severe COVID-19 disease (see lines 156-173). The genes differentially expressed in severe vs. mild are highlighted in red in Figure 3, and this is indicated in the figure legend.

Reviewer 1 - The gating strategy is shown in Supplementary Figure 6 instead of Supplementary Figure 7 (lines 233-234).

RESPONSE - We have corrected the reference in the text to now refer to Supplementary Figure 6.

Reviewer 2 - This is very solid work, with high quality and valuable data generated on whole genome ATAC-seq and Capture C on a number of primary cell lines. This data is then used for a targeted analysis of a particular GWAS associated region. The analysis of the SARs-Cov19 associated regions is also solid, with appropriate analysis of the independent signals and proxy SNPs. There is also relevant and accurate bioinformatic analysis performed from public data sources. The work is presented very clearly and is very well written. I think to demonstrate a clear advance from the GWAS signals to putative causal genes in specific cell types, over and above publicly available data on expression, TFBS and gene ontology and the in-house whole genome generated data on activity/interactions, at least one of the interactions needs to be validated as being functional in a particular cell type.

RESPONSE - We thank the reviewer for their appreciation of the value of our study, and we agree that functional follow up would greatly improve the impact of the manuscript. Indeed, we now report on the role of GART, one of the novel COVID-19 variant-connected genes identified in this study, in the T cell-dependent generation of antibody-producing B cells in a human tonsillar organoid model. In a new Figure 6, we show that lometrexol, a specific pharmacologic antagonist of GART, abrogates T cell- dependent induction of follicular antibody-secreting plasmablasts in a highly physiologic human tonsillar organoid model. This shows that GART has a previously unappreciated role in T cell-B cell collaboration for the production of class-switched, high affinity antibody, which is a process linked to COVID-19 disease severity. These new results demonstrate that our V2G mapping of genes involved in COVID severity has identified a novel target whose activity could be promoted for better anti-viral humoral immune responses or inhibited as a potential treatment for systemic/Ab- mediated autoimmune disease.

**2^nd^ round**

**Reviewer 1**

Thanks to the authors for addressing my points. Some additional minor comments for improving the clarity:

1. COVID-19 GWAS data - I’m still confused. First, please specify the data freeze for COVID-19 GWAS by the COVID-19 GWAS Host Genetics Initiative in the Methods section. Second, in the Methods section you mention that you curated the lead SNPs from the recent COVID-19 severity GWAS from the COVID-19 HGI and that 13 sentinel SNPs represented 8 independent signals (lines 392-393; on line 105 you mention 6 independent loci – is this an error in the methods section?). Could you please clarify which of the COVID-19 phenotypes (critical illness, hospitalized, or reported infection) by the COVID-19 HGI is used as proxy for COVID-19 severity in your study? Also, COVID-19 HGI based on the freeze 5 data reported 13 independent loci across the three phenotypes. Are those 13 independent loci the same as your 13 sentinel SNPs (which were further narrowed done to 6 independent signals - how?)? If yes, then you are assessing all the loci associated to COVID-19 and not just the loci associated with more severe outcomes of COVID-19.

2. Lines 126-127: “rs10490770 is the highest signal located on chromosome 3” – the highest signal -> the strongest signal.

3. Figure 2C uses “naiveT” (as the other figures panels of Figure 1 and Figure 1), but Figure 1C still uses “naïve CD4”.

4. Figure 3 – while I understand that two different comparisons are discussed in the main text (infected vs uninfected and mild vs severe), I’m still confused about the highlighting of the genes. Six genes (IFNAR2, OAS1, OAS3, PAXBP1, GART, TNFAIP8L1) are claimed to exhibit severity-associated patterns of expression (line 140) and are highlighted in red in Figure 3 as indicated in the figure legend. However, only five genes are highlighted in Figure 3. Should IFNAR2 be highlighted as well?

**Authors’ response**

Reviewer #1: Thanks to the authors for addressing my points. Some additional minor comments for improving the clarity:

1. COVID-19 GWAS data - I’m still confused. First, please specify the data freeze for COVID-19 GWAS by the COVID-19 GWAS Host Genetics Initiative in the Methods section. Second, in the Methods section you mention that you curated the lead SNPs from the recent COVID-19 severity GWAS from the COVID-19 HGI and that 13 sentinel SNPs represented 8 independent signals (lines 392-393; on line 105 you mention 6 independent loci – is this an error in the methods section?). Could you please clarify which of the COVID-19 phenotypes (critical illness, hospitalized, or reported infection) by the COVID-19 HGI is used as proxy for COVID-19 severity in your study? Also, COVID-19 HGI based on the freeze 5 data reported 13 independent loci across the three phenotypes. Are those 13 independent loci the same as your 13 sentinel SNPs (which were further narrowed done to 6 independent signals - how?)? If yes, then you are assessing all the loci associated to COVID-19 and not just the loci associated with more severe outcomes of COVID-19.

We thank the reviewer for the suggestions to improve clarity. The sentence describing the number of input SNPs is an error that remained from the prior analysis of Pairo- Castineira as mentioned in the last review. We apologize for the confusion and have clarified the methods 401-402. We have now clarified that we are using Freeze 5 in the Methods section. For the sentinel SNP we selected the significant associations with critical illness and with the suggested phenotypic impact as disease severity which yield the six independent signals used in our analysis (derived from supplementary table 2 of the flagship COVID-19 Host Genetics Initiative, 2021 paper). We did not include the three signals at suggestive P values.

2. Lines 126-127: “rs10490770 is the highest signal located on chromosome 3” – the highest signal -> the strongest signal.

We have corrected the terminology.

3. Figure 2C uses “naiveT” (as the other figures panels of Figure 1 and Figure 1), but Figure 1C still uses “naïve CD4”.

We have now changed Figure 2C to NaiveT. We have also corrected supplementary figure 3 which also was labeled NaiveCD4.

4. Figure 3 – while I understand that two different comparisons are discussed in the main text (infected vs uninfected and mild vs severe), I’m still confused about the highlighting of the genes. Six genes (IFNAR2, OAS1, OAS3, PAXBP1, GART, TNFAIP8L1) are claimed to exhibit severity-associated patterns of expression (line 140) and are highlighted in red in Figure 3 as indicated in the figure legend. However, only five genes are highlighted in Figure 3. Should IFNAR2 be highlighted as well?

We have corrected the figure to now also highlight IFNAR2.
